# Payment of Fees for Attendance in Midwives' Cases

**Published:** 1910-02-19

**Authors:** A. Adair Dighton

**Affiliations:** Warwick House, Portland Street, Cheltenham


*To the Editor of* The Hospital.

### PAYMENT OF FEES FOR ATTENDANCE IN MIDWIVES' CASES.

*To the Editor of* The Hospital.

Sir,—I was sent a form, headed by Glos. County Council, signed by District Certified Midwife, on November 7, 1909, for assistance at a midwife's case. I went, and delivered with forceps. I have applied to the medical officer of health for Gloucestershire, and he has referred me to the Winchcombe Board of Guardians, and they decline to pay. To whom am I to look for my well-earned fee—£2 2s.? The village is three and a-half miles off. I think it best to work through The Hospital, as I have no doubt others are anxious about their fees. Yours truly,

A. Adair Dighton.

Warwick House, Portland Street, Cheltenham.
January 23, 1910.

